# “I had to work it out for myself”: opportunities for primary care to fill a void for early-onset breast cancer survivors

**DOI:** 10.1007/s11764-026-02001-9

**Published:** 2026-03-18

**Authors:** Sarah J. Fadem, Anna Maniaci, Katie A. Devine, Denalee M. O’Malley, Jennifer R. Hemler, Gianna Holover, Shawna V. Hudson, Benjamin F. Crabtree

**Affiliations:** 1 Department of Family Medicine and Community Health, Research Division, Rutgers Robert Wood Johnson Medical School, 303 George St., Matrix Plaza I Building, 3rd Floor, New Brunswick, NJ 08901 USA; 2 Rutgers Cancer Institute of New Jersey, 195 Little Albany St., New Brunswick, NJ 08901 USA

**Keywords:** Early-onset breast cancer, Survivorship, Primary care, Patient-provider communication

## Abstract

**Purpose:**

Breast cancer incidence in women under 50 (early-onset) is steadily increasing. Primary care clinicians can play a role in managing late and long-term treatment effects for these women, who face decades of survivorship and are coping with a life-stage discordant illness. This study describes early-onset breast cancer survivors’ experiences with primary care.

**Methods:**

Semi-structured interviews were conducted with early-onset breast cancer survivors (*N* = 16). Iterative, inductive thematic analysis was used to identify patterns in experiences with primary care.

**Results:**

Participants were on average 58.4 years old and 17 years post-diagnosis (*M*_age_ at dx = 41.4). Relationships with current primary care clinicians were generally short (mean = 5.8 years; median = 1.5 years). Many survivors lacked continuity, being either disconnected from oncology (*N* = 6) or seeing an oncology specialist not on their initial treating team (*N* = 7). Despite high rates of late/long-term treatment effects, including cardiovascular issues (*N* = 10), premature menopause (*N* = 10), and pain (*N* = 7), participants rarely turned to primary care clinicians for support. This fragmentation was often normalized by survivors, who felt they had to self-advocate for their own long-term health management.

**Conclusions:**

Early-onset breast cancer survivors navigate survivorship in a fragmented healthcare system and bear the burden of coordinating their own care. Dynamic information support tools are needed to empower survivors to communicate their cancer history and connect symptoms to cancer-related issues in primary care settings.

**Implications for Cancer Survivors:**

As clinical continuity is limited over decades of survivorship, early-onset cancer survivors need resources that bridge the gap between their cancer history and current primary care management.

**Trial registration:**

Registered with ClinicalTrials.gov on June 2, 2022: NCT05400941, https://clinicaltrials.gov/study/NCT05400941

## Background

The rate of breast cancer diagnoses in women under the age of 50, often referred to as early-onset breast cancer, has been steadily increasing over the past two decades [1, 2]. In 2024, an estimated 60,000 women under 50 years old were diagnosed with breast cancer in the USA [3]. This growing population has unique challenges compared to older survivors, as they often present with more aggressive tumors with poorer prognoses and consequently receive more aggressive treatments [4–9]. Still, these survivors are likely to live decades beyond their diagnosis and potentially face ongoing sequelae of treatment and accelerated aging [10, 11]. This includes cardiovascular disease [12–15], premature menopause [16–19], cognitive dysfunction [20, 21], and psychological distress [22–25]. These survivors are at increased risk of age-related comorbidities [26], including hypertension, weight gain [27, 28], hyperlipidemia [29–32], and osteoporosis [33, 34] years after treatment. These health challenges frequently emerge during critical life stages, while survivors are building careers and caring for young children [35–38], requiring them to balance issues of premature aging with career or caregiving responsibilities.

Young women with breast cancer are also at increased risk of secondary cancers. This risk is linked to cumulative treatment toxicity and the genetic mutations common in this population [39]. Ongoing oncology surveillance is important for monitoring recurrence; however, the unique risk of this population for late effects and secondary cancers, as well as the elevated risk of cardiovascular disease and metabolic syndrome, can benefit from the longitudinal relationship and comprehensive screenings offered by primary care. Much of the care that cancer survivors need long-term, including surveillance for recurrence or secondary cancers, control of non-cancer comorbidities, and identification and management of treatment-related side effects, is a core function of primary care clinicians [40, 41]. Evidence suggests that primary care involvement post-cancer diagnosis can lead to optimal comorbidity management [42–46]. Primary care clinicians are experienced in providing care to patients with multiple comorbidities and prioritizing among conditions [47, 48], an unfortunate reality for many cancer survivors facing ongoing sequelae of cancer treatments [49].

As the number of long-term cancer survivors continues to grow, with an estimated 40% of all survivors being 15 or more years from diagnosis by 2040 [50], primary care involvement in cancer survivorship care will become more critical. The growing number of long-term survivors will outpace the supply of oncologists available, making an oncology-only model unsustainable [51, 52]. Despite this need, the current healthcare infrastructure often creates a disconnect between this population and primary care. A recent study of young adult breast cancer survivors 7 years post-diagnosis found that while 92% reported having a primary care clinician, more than half saw only an oncology provider for cancer-related follow-up [53]. Being younger at cancer diagnosis is also associated with lower odds of having annual primary care visits [54].

Guidelines from leading cancer organizations encourage primary care involvement in cancer survivorship care, which is defined as assessing and mitigating the impact of cancer and its treatment [55]. Recent National Standards for Cancer Survivorship Care include coordination with primary care as a key indicator of care quality [56]. However, significant barriers, such as unclear roles for primary care clinicians and poor communication between primary care clinicians and oncologists [57–59] often prevent this from becoming a reality, and survivorship care remains fragmented [60–62]. Post-diagnosis, cancer patients commonly see multiple clinicians from different specialties [63], but a lack of system-level infrastructure [64–67] and evidence-based recommendations for implementing shared care models [68] limit care coordination. In this fragmented system, care gaps are filled by survivors or not addressed at all [69–71].

This gap between recommendations for primary care involvement and the current reality is also reflected in both patient and clinician perspectives. Survivors may prefer oncology-based follow-up, driven by the trust and connection they have built with the oncology team and their desire for reassurance that their cancer has not reoccurred [72, 73]. While survivors may value the holistic approach of primary care clinicians, they often see them as less knowledgeable about cancer follow-up or late/long-term effects of cancer treatment [72, 74, 75]. These perceptions may be mirrored by primary care clinicians themselves, who report a lack of cancer-specific knowledge as a significant challenge to caring for cancer survivors, along with confusion over who is responsible for which aspects of care between primary care and oncology [76], and low confidence in providing survivorship care [77].

While the barriers to primary care involvement in survivorship care are well documented, few studies have examined the perceptions of early-onset breast cancer survivors on the role of primary care. This population stands to significantly benefit from primary care involvement in survivorship due to the long survivorship period and high potential for late/long-term treatment effects from aggressive treatments. This study sought to understand how early-onset breast cancer survivors experience primary care involvement in their survivorship care. Specifically, we aimed to understand their relationships with primary care clinicians and the management of their late/long-term effects from cancer and its treatment. Our goal is to identify opportunities for future interventions to improve survivorship care for this growing population.

## Methods

### Study design

The interviews conducted and analyzed for this study were collected as the baseline assessment of breast cancer survivors’ experiences with primary care as part of a larger study. The parent study aimed to refine and test primary care–informed strategies to integrate breast cancer survivorship evidence in primary care practices in a large regional health system in the Northeastern United States. The protocol of the parent study was reported elsewhere [78]. All procedures were approved by the Rutgers Institutional Review Board. The study is reported according to the Standards for Reporting Qualitative Research [79].

### Participants and recruitment

A purposive sample of patients with a breast cancer history documented in the electronic health record (EHR) was recruited from the primary care panels of practices participating in the parent study. Participants were identified through the patient portal based on initial eligibility criteria: (1) minimum age of 18; (2) documented primary care clinician connected to the parent study; (3) documented history of breast cancer using International Classification of Diseases (ICD) codes; and (4) consent to be contacted for research. Participants were then sent an automated message through the patient portal with a description of the study and a prompt to indicate interest in participation. Interested participants were asked to complete an online eligibility screener. Those who did not complete the screener were contacted directly by phone to ascertain interest and conduct the screening verbally. Patients were eligible if they had (1) completed breast cancer treatment; (2) spoke English; (3) had a visit with their primary care clinician in the past 2 years; and (4) had at least one visit with their primary care clinician post-diagnosis. Patients were excluded if they had a stage IV diagnosis, were currently undergoing treatment, or had documented physical or mental health conditions that precluded their ability to provide informed consent or participate in an interview (e.g., dementia diagnosis). Individuals were purposefully selected from the list of interested participants to be invited for an interview to maximize variation in experiences across demographic and clinical characteristics [80]. The selected participants were then contacted again via phone and email to reconfirm interest in interview participation. Participants were emailed the consent form and offered the option to complete the interview by phone or video call. Verbal consent for participation and recording was obtained at the start of each interview. For the parent study, 25 participants were interviewed; however, this analysis focuses only on the subset of participants who are early-onset breast cancer survivors (e.g., diagnosed before age 50) (*N* = 16).

### Data collection

Semi-structured in-depth interviews were designed to assess the following constructs: primary care clinician relationships, primary care involvement and role during breast cancer diagnosis and treatment, non-cancer comorbidity management during cancer treatment, primary care clinician assessment of late and long-term treatment effects, patient portal experiences, how clinicians on their care teams coordinate, and patient sources of information. During interviews, participants were asked to review a list of potential late- or long-term effects of cancer treatments informed by the American Cancer Society/American Society of Clinical Oncology (ACS/ASCO) guidelines (e.g., cardiovascular issues, brain fog, fatigue, pain/neuropathy, premature menopause/hot flashes, and sexual health), and whether their primary care clinician had discussed any of these symptoms in relation to their breast cancer history. Participants were asked for suggestions on how primary care clinicians could improve care related to breast cancer history. Interviews took place virtually on the Zoom platform and were recorded and transcribed verbatim. Interviews were conducted by two research assistants trained in qualitative interview techniques, one with a background in public health and the other in psychology. The latter research assistant (GH) also participated in the analysis process. Participant demographic information was collected through self-report at the end of the interview. Interviews took place between December 2024 and March 2025 and lasted approximately 40 min. Participants received $25 as compensation for their time.

### Data analysis

The team engaged in an iterative, inductive style of thematic analysis to identify patterns in the data [81] using Atlas.ti software that involved reading, rereading, and summarizing the data. The analysis team consisted of four analysts (SF, BC, AM, GH) and was overseen by the two senior analysts (SF, BC) with experience in qualitative methods, primary care practice transformation, cancer survivorship care delivery, and care coordination. An initial read of 5 transcripts was used to develop a preliminary codebook based on patterns identified, which centered on how interviewees understood and made sense of their experiences around late/long-term effects and the role of primary care in their survivorship experience. The team independently reviewed transcripts and met regularly to discuss identified codes/categories and emergent patterns in the data. These initial codes were applied across transcripts. Coding discrepancies were resolved through discussion to reach consensus among the team members.

Given our focus on experiences with primary care, we extracted all text segments related to patients’ perceived relationship to primary care providers and their descriptions of the primary care provider’s role in their survivorship care. This was a heavily reflective process in which team members kept memos and met weekly to discuss patterns and next steps for the analysis. The larger research team reviewed the results of these analyses to finalize the interpretation and resulting themes.

### Reflexivity

The research team members had diverse backgrounds in health communication, medical anthropology, health psychology, social work, and primary care practice transformation. This multidisciplinary approach allowed for triangulation of perspectives during data interpretation. Research team meetings were used as forums to challenge assumptions and ensure the themes were driven by participants’ described lived experiences.

## Results

### Participant characteristics

Participants (*N* = 16) had a mean age at time of interview of 58.4 years old, and they were, on average, 17 years post-diagnosis. Participants’ mean age at diagnosis was 41.4 years old (see Table 1 for participant characteristics). Majority of participants (*N* = 15; 93.75%) reported experiencing at least one late- or long-term effect of their cancer treatment, most commonly cardiovascular issues (*N* = 10; 62.5%), premature menopause (*N* = 10; 62.5%), and pain (*N* = 7; 43.75%). Despite the long survivorship period, most participants (*N* = 10, 62.5%) were actively seeing an oncology clinician. Among the 10 survivors attached to cancer care, most (*N* = 7; 70%) were no longer seeing their original oncology team. This lack of continuity was observed among primary care relationships as well. Few participants (*N* = 2; 12.5%) retained the same primary care clinician they had when they were diagnosed with breast cancer. Three survivors reported having had strong relationships with their primary care clinician at diagnosis, but those clinicians had since retired. Few participants (*N* = 3; 18.75%) did not have a primary care clinician when they were diagnosed with breast cancer. The average length of primary care clinician relationships was 5.8 years (median = 1.5 years; calculated for *N* = 15 as one participant provided an estimated range).

**Table 1:** Participant characteristics

	*N* (%)
Age at diagnosis	
20–29 years	1 (6.25)
30–39 years	3 (18.75)
40–49 years	12 (75)
Current age	
40–49 years	3 (18.75)
50–59 years	7 (43.75)
60–69 years	3 (18.75)
70–79 years	3 (18.75)
Time from diagnosis	
3–5 years	1 (6.25)
6–10 years	5 (32.25)
> 10 years	10 (62.5)
Treatment received	
Surgery only	4 (25)
Surgery, chemotherapy	3 (18.75)
Surgery, radiation	3 (18.75)
Surgery, radiation, chemotherapy	5 (31.25)
Radiation only	1 (6.25)
Adjuvant hormone therapy	9 (56.25)
Race	
White	12 (75)
African American/Black	2 (12.5)
Asian	1 (6.25)
Mixed Race	1 (6.25)

We categorized participants into two groups based on the length of their relationship with their current primary care clinician: (1) short-term group (relationship ≤ 2 years; *N* = 8) and (2) long-term relationship (> 2 years; *N* = 8) (Table 2). This threshold was determined inductively during the analysis; we initially examined relationships based on a 5-year cutoff, but observed that participants with relationships as short as 3 years described a distinct sense of longitudinal support. Beyond the 2-year mark emerged as the inflection point where survivors began to describe navigational support, contrasting with the fragmented experiences of those in newer relationships. The reasons for the short-term relationships were nearly all systemic or practical (e.g., insurance changes, clinician retirement, or patient relocation). This lack of continuity with both primary care clinicians and cancer care teams created challenges for many survivors.

**Table 2:** Characteristics of short- and long-term primary care relationships

	Short-term primary care relationship (≤ 2 years)	Long-term primary care relationship (> 2 years)
***N***	**8**	**8**
Length of survivorship (years)		
Mean	14.69	19.13^1^
Median	10.5	18
Range	6–25	4–34
Length of current PCP relationship (years)		
Mean	.86	11.14
Median	.875	10
Range (years)	0–2^2^	3–30
Still seeing a member of their original treating oncology team (*N* = 3)	2	1
Seeing a new oncology clinician (not the one who treated them) (*N* = 7)	4	3
No longer in routine oncology follow-up (*N* = 6)	2	4

^1^Missing data on exact relationship length for one participant, but patient stated she had been seeing her primary care clinician for “a very long time”

^2^One participant had recently had her primary care clinician retire and had been reassigned to another clinician in the practice, whom she had yet to meet

**Fig. 1:**
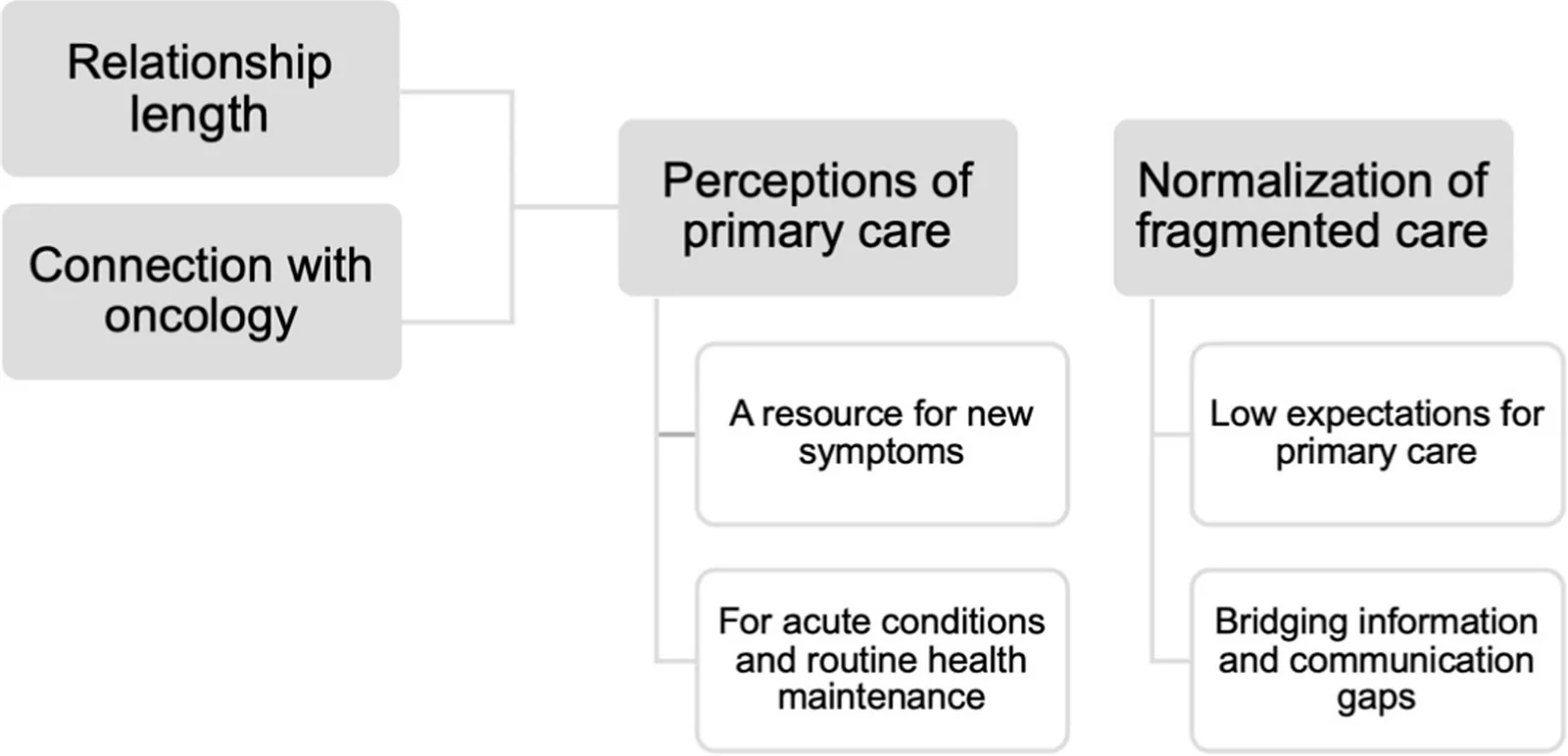
Visualization of themes

Analysis revealed (1) variations in survivor perceptions of primary care, influenced by relationship length and status of connection with oncology, and (2) the normalization of fragmented care. Participants either saw primary care as either a resource for new symptoms that could possibly be related to breast cancer history, or they perceived primary care as reserved for acute conditions or routine health maintenance. Long-term relationships with primary care often mitigated effects of this fragmentation, but not always. For many survivors, fragmentation became normalized, leading to lower expectations of services offered by primary care clinicians to address their concerns. The burden of this fragmentation fell on survivors (Fig. 1).

### Perceptions of primary care

While the majority of survivors reported a positive relationship with their primary care clinicians, these relationships were limited in providing survivorship-specific care. This gap was exacerbated by a lack of clinical continuity as most survivors were navigating their long-term health with a relatively new group of clinicians, either on the oncology or primary care side. No survivor was still seeing both the primary care clinician and the oncologist that they were seeing when first diagnosed. Primary care clinicians consistently were involved in routine cancer screenings, such as coordinating mammograms, but survivors did not view them as being the primary manager for their long-term treatment-related symptoms, regardless of relationship length.

#### Primary care as a resource for new symptoms

Nine participants reported going to their primary care for symptoms that could be related to their breast cancer history (*N* = 7 long-term primary care relationships; *N* = 2 short-term primary care relationships). Four of these participants were disconnected from oncology and four had a new oncologist that was not their original treating oncologist. The one participant who was still connected with her original oncologist reported going to her relatively new primary care clinician for possible sequelae after her oncologist failed to address her concerns:

I feel that when I bring up some of the concerns that I have with some of the things that I still face from treatment and all of the side effects long term, when I bring it up to my oncologist, he’s like, that’s kind of not my problem. When I bring it up to my primary… it’s always, oh, you have to go here or you have to go here type of things… I have, still, a lot of brain fog, and I also have issues with sexual intercourse and things like that, like I have the vaginal atrophy, and it has been seven years since I’ve been with my husband, and it’s like my doc – my oncologist is like talk to your OB-GYN, my OB-GYN was like, eh, you’ve had a hysterectomy, I don’t know what we can do, and we can’t give you hormones. So it’s – I feel like you – oh, you just have to deal with it. And it’s like, I’m 44, and I don’t want to really deal with it. (10-year breast cancer survivor still seeing her original oncologist; she has been with her current primary care clinician for 1 year)

The concerns these nine participants brought to their primary care clinicians were not always framed as being related to their cancer history. For instance, one participant (a 10-year survivor who had been with her primary care clinician for five years) was one of a handful of participants who had seen their primary care clinicians because of fatigue. Her clinician ordered bloodwork and ultimately suggested that the patient start taking vitamins, with no mention of the possible connection to breast cancer history. Participants reported that while primary care clinicians often screen for conditions commonly seen in primary care that are also known treatment effects (i.e., cardiovascular disease and depression), they rarely initiated conversations describing the possible link to the patient’s cancer history.

#### Compartmentalization of roles

Independent of relationship length, participants largely described primary care as being for acute conditions or health maintenance. Many survivors, particularly those with short-term primary care relationships, lacked clarity on if the primary care clinician can or should be involved in survivorship care and consistently described the burden of navigating the fragmented health system on their own.

A common sentiment for participants who did not report going to primary care for possible late/long-term effects (*N* = 7) included having specialists “for everything else.” Of these seven, six had short-term primary care relationships; the sole participant in this group with a long-term primary care relationship was the only participant in this study with both a long-term primary care relationship and a connection to her original treating oncology team. One 25-year breast cancer survivor still connected with her breast surgeon described feeling “very comfortable” with the primary care clinician she had been seeing for the last 9 months but explained that “she knows I’m being followed by these other people.”

Despite viewing their primary care clinicians positively for routine care, those with short-term primary care relationships often described a system where they had to act as their own advocates. The primary care clinician was another silo rather than a central point of care. This was described by one survivor who had just switched to a new primary care doctor after her previous primary care clinician retired:

I felt like I had the oncologist, you know, where we see him every year for ten years. He was really my primary at that time until, until a couple of years ago where I just said, okay, I’m, you know, I’m fine. I didn’t see him anymore. Now… I’m kind of just going to primary once a year. And there’s no discussion about it…It’s like everything is separate. It’s like every doctor treats a different part of your body. (24-years post-diagnosis breast cancer survivor; discharged from oncology and her long-time primary care clinician had recently retired)

#### Long-term primary care relationships mitigating effects of fragmentation

While fragmentation was experienced by survivors with both short- and long-term primary care relationships, long-term primary care relationships provided a safety net that newer relationships lacked. Survivors with primary care relationships longer than 2 years often described feeling that they could turn to their primary care clinician for any issue they were not sure how to manage on their own. As one participant, a 21-year survivor discharged from oncology who has been seeing her primary care clinician for 11 years, put it, “It’s nice to have someone who centrally knows everything.” These survivors experienced more integrated care and received trusted referrals from a primary care clinician they saw as a knowledgeable and reliable first point of contact. As one survivor put it, her primary care clinician is “holding the umbrella and he’s referring me out as I need”:

He can help really to navigate really any of my care… he called me and he said, I want you to see the cardiologist, but in the meantime, I’m gonna put you on a statin, and then let him know that you started on the statin…it wasn’t like he said, well, you’re gonna have to wait to see the cardiologist, he took – he takes the initiative to do what needs to be done and that’s why I like him. (11-year survivor who still sees her breast surgeon and who has been seeing her primary care clinician for 10 years)

This survivor noted that her primary care clinician asks her if she has seen her breast surgeon, functioning as “just like a touch point to say, how is it going and do you need anything from me?” Even survivors with stable, long-term primary care relationships reported that the health system remained fragmented.

### Normalization of fragmented care

All survivors, even those who described being frustrated or let down by their care team, generally viewed their primary care clinicians positively. The expectations of primary care clinicians seemed to be met because their expectations were limited to acute care and routine tasks such as following up on screenings, doing annual physicals, and providing referrals when needed. While primary care clinicians were involved in regular screenings like mammograms, they rarely initiated conversations linking current health issues to past cancer treatment.

#### Low expectations

A 34-year survivor who had been seeing her primary care clinician for 3 years and thinks he is “the best” pointed out that she does not bring up some of the issues she faces after treatment to her primary care doctor because she is still seeing an oncologist (though not the one who originally treated her). She explained her reasoning simply, “because he’s not my breast cancer doctor.” Still, when asked if she wished her primary care doctor would ask more questions about her cancer history, she responded, “He’s the perfect guy. Do I wish it was more? Always more when it comes to breast cancer because it happened to me when I was very young.”

While some survivors maintained satisfaction by lowering their expectations, others acutely felt the loss of the holistic care they received during their active treatment. One survivor, who worked in healthcare administration, reflected on the differences between the primary care clinician she was seeing when she was diagnosed, who acted as the “captain of the ship” during her treatment but had since retired, and the primary care clinicians she had seen since:

When you’re first diagnosed, there’s so many different doctors that you see and [my first primary care clinician] was, for lack of a better word, captain of the ship… she didn’t want my care to be siloed like I’m seeing the radiation oncologist, they’re only looking at this and the breast surgeon only looking at this… we’re in a different healthcare environment unfortunately now and so my expectations and my experience may be very different… being on the other side of that, where you had someone that would hold your hand and spend 25 minutes and allow the office staff to be able to help was just really an incredible blessing. And I don’t know that if that is really feasible or practical in this day and age because we're just kind of all squeezed for our resources. (11-year survivor still seeing oncology who has been with her current primary care clinician for 9 months)

#### Bridging communication and information gaps

Because of systemic fragmentation, survivors found themselves acting as care coordinators navigating the gaps between specialties. One survivor described how her experience navigating this fragmented care system challenged her understanding of the ideal primary care clinician and led her to lower her expectations for what primary care could provide:

My experience has been that I had to be my own medical advocate, and I had to work it out for myself, amongst various doctors… Theoretically what is supposed to happen is you were supposed to have an internist who was the door to all of these various other specialists and care. And it’s just not reality… I don’t see it happening anytime soon (21.5-year survivor discharged from oncology who has been with her current primary care clinician for 1 year)

This burden also included information transfer, as one survivor told the story of her primary care clinician referring her to an orthopedist, then relying on the patient to ensure the specialist communicated back, saying to “make sure that [the orthopedist] calls me or that he communicates with me about the results here, because otherwise, she said, I’ll fall out of the chain of information and I'll never know.” For early-onset survivors who face decades of complex survivorship, self-advocacy often feels necessary to bridge care gaps.

## Discussion

Most of the early-onset breast cancer survivors in this study were being cared for by clinicians who had not been a part of their initial diagnosis and treatment, reflecting the lack of continuity with both primary care and oncology clinicians. Our participants experienced a significant gap between their survivorship duration and continuity with their primary care clinicians (17 vs. 5.8 years). Because of this fragmentation, no single clinical team had a longitudinal perspective on these participants’ health, putting a greater burden on survivors to advocate for themselves to receive treatment for their late/long-term treatment effects. The reliance on self-advocacy can exacerbate inequities, as survivors who cannot actively petition for their care may not receive the care they need.

This fragmentation likely has many factors. Compared to older women, younger women may lead a less stable lifestyle and be more engaged in their career or family obligations, as evidenced by participants changing primary care clinicians after moving or insurance changes. While there are multiple transitions during survivorship that can lead to addition or loss of clinicians on a patient’s care team, this may be more important for younger survivors whose life circumstances are more likely to change. The gaps in care for these survivors of early-onset breast cancer were not caused by a single failure but fragmentation over a long survivorship period. High clinician turnover is not surprising for a 17-year cancer journey; however, our health system does not have effective procedures and tools for managing this reality.

Our participants described positive, trusting relationships with their primary care clinicians, yet they rarely turned to them for the late/long-term treatment effects that many experienced. This aligns with previous literature suggesting that survivors rate primary care clinicians highly in providing holistic, patient-centered care and psychosocial support [74, 82] while also having low expectations of primary care’s role in survivorship, due to the limited resources and time of primary care clinicians [83]. This results in a system where fragmentation is normalized, and patients take on the role of coordinator and advocate; they are not just “lost in transition” from oncology but in a state of interrupted continuity with no anchor. Primary care clinicians can act as the central point in survivorship care rather than another in a list of clinicians of various specialties if offered tools that leverage their idealized function as the primary coordinator who identifies issues best served by specialists versus those that can be managed in primary care [47].

Importantly, late/long-term effects persist or arise years after treatment. For example, the risk of CVD-related mortality manifests about 7 years after breast cancer diagnosis [84]. What makes care fragmentation especially damaging for early-onset breast cancer survivors is the combination of their young age at diagnosis (often pre-menopausal) and long survivorship period. Just as they become increasingly at risk of serious treatment effects, clinician memory of that treatment is low. Many primary care clinicians are not trained to see these issues as within their domain. The long-term survivors interviewed were in the stage of survivorship when late effects of treatment are known to emerge, and half were seeing relatively new primary care clinicians. Nearly all the women interviewed experienced symptoms of late/long-term treatment effects; most of these health issues, such as cardiovascular conditions and pain, are commonly treated by primary care clinicians in patients with or without cancer history. Still, younger women may not be screened for these common conditions of aging if their cancer history is not known. Ensuring that primary care clinicians are aware of cancer history and the potential for accelerated aging can facilitate the identification of these issues that may otherwise be unassessed in a younger population.

### Implications for cancer survivorship care

As more women are diagnosed with breast cancer before age 50, primary care clinicians may see an increase in patients with early-onset breast cancer history. Rather than placing another requirement on already strained primary care clinicians, we need a systemic solution. The high rate of discontinuity requires that cancer history is visible and actionable for all clinicians that join a patient’s care team. Consolidation in health systems has increasingly encouraged continuity with a “practice” rather than a specific physician, increasing the need for data sharing. Potential leverage points include primary care intake processes and electronic health records (EHRs). Intake forms could systematically ask all patients about any personal cancer history, especially young women as they carry a higher burden of cancer diagnoses than young men [85], and capture this information in the EHR. This technology could then flag for patients at high risk of late effects such as CVD and address barriers to information sharing, facilitating risk-stratified care and enabling primary care clinicians to provide surveillance for a vulnerable and growing population [86]. EHRs that gather all relevant medical information can be particularly impactful for cancer survivors. Health systems can implement EHR algorithms to flag early-onset survivors for specific cardiovascular and metabolic risk assessments, prompting conversations during routine visits. Establishing designated care coordinators that facilitate handoffs between primary care and oncology, including when patients transition to new clinical teams, could also mitigate issues with continuity.

Further, the fact that many of these long-term survivors were still seeing an oncology clinician presents another opportunity for improved coordination. A recent study found that long-term survivors with regular oncology visits were more likely to have regular primary care visits [54]. While oncology visits do not guarantee continuity, they may encourage patients to maintain broader healthcare engagement. If oncologists encourage continued connections with primary care, patients may more consistently tap into connections with primary care as they transition away from oncology or experience clinician turnover. Patient education materials from oncology could more clearly outline potential late effects and emphasize the role of primary care for ongoing survivorship issues. There is also an opportunity for advocacy groups to work with oncologists and primary care clinicians to provide patients with the tools to advocate for themselves and guide conversations with their clinicians in the years after treatment [87].

This study has several limitations. The sample size is of 16, with most participants being White, which may limit generalizability. Participants were recruited from a single health system and had a documented primary care clinician, which may not reflect the experiences of survivors without consistent primary care, uninsured patients, or those seen in federally qualified health centers. Finally, the interview guide was not designed specifically for early-onset survivors, so some nuances of their experiences may be missing; however, the fact these themes emerged strongly without direct prompting suggests these issues are central to survivors’ experiences. Future studies should look intentionally at these areas.

## Conclusion

Early-onset breast cancer survivors currently navigate a fragmented healthcare system where the burden of care coordination often falls on the patient. To support this growing population, we must move beyond static survivorship care plans towards dynamic information support for survivors in the years after treatment. This shift requires collaboration between oncology, advocacy groups, primary care, and health information technology teams to empower patients and primary care clinicians to recognize and manage long-term sequelae of cancer treatment.

## Data Availability

Interview transcripts are not available to protect participant confidentiality.
